# Effect of Amine, Carboxyl, or Thiol Functionalization of Mesoporous Silica Particles on Their Efficiency as a Quercetin Delivery System in Simulated Gastrointestinal Conditions

**DOI:** 10.3390/foods13081208

**Published:** 2024-04-16

**Authors:** Alexis Matadamas-Ortiz, Juan F. Pérez-Robles, Rosalía Reynoso-Camacho, Silvia L. Amaya-Llano, Aldo Amaro-Reyes, Prospero Di Pierro, Carlos Regalado-González

**Affiliations:** 1 Departamento de Investigación y Posgrado en Alimentos, Facultad de Química, Universidad Autónoma de Querétaro, C.U., Cerro de las Campanas s/n, Col., Las Campanas, Querétaro 76010, Qro., Mexico; amatadamas717@alumnos.uaq.mx (A.M.-O.); rrcamachomx@yahoo.com.mx (R.R.-C.); samayal@uaq.mx (S.L.A.-L.); aldo.amaro@uaq.edu.mx (A.A.-R.); 2 Unidad Querétaro, Centro de Investigación y Estudios Avanzados del IPN, CINVESTAV, Libramiento Norponiente No. 2000, Fracc. Real de Juriquilla, Querétaro 76230, Qro., Mexico; jfperez@cinvestav.mx; 3 Department of Agricultural Sciences, University of Naples Federico II, Via Università 100, 80055 Portici, Italy; prospero.dipierro@unina.it

**Keywords:** functionalized mesoporous silica, quercetin, simulated gastrointestinal digestion, quercetin delivery system

## Abstract

Quercetin (Q) dietary supplements exhibit poor oral bioavailability because of degradation throughout gastrointestinal digestion (GD), which may be overcome using mesoporous silica particles (MSPs) as an oral delivery system (ODS). This study aimed to elucidate the effect of the functionalization of MSPs with amine-(A-MSP), carboxyl-(C-MSP), or thiol-(T-MSP) groups on their efficiency as a quercetin ODS (QODS). The type and degree of functionalization (DF) were used as factors in an experimental design. The Q-loaded F-MSP (F-MSP/Q) was characterized by gas physisorption analysis, loading capacity (LC), and dynamic light scattering and kinetics of Q release at gastric and intestinal pHs. Antioxidant capacity and Q concentration of media containing F-MSP/Q were evaluated after simulated GD. A-MSP showed the highest LC (19.79 ± 2.42%). C-MSP showed the lowest particle size at pH 1.5 or 7.4 (≈200 nm). T-MSP exhibited the maximum Q release at pH 7.4 (11.43%). High DF of A-MSP increased Q retention, regardless of pH. A-MSP preserved antioxidant capacity of Q-released gastric media (58.95 ± 3.34%). Nonetheless, MSP and F-MSP did not protect antioxidant properties of Q released in intestinal conditions. C-MSP and T-MSP showed essential features for cellular uptake and Q release within cells that need to be assessed.

## 1. Introduction

Quercetin (Q), or “3, 3′, 4′, 5, 7-pentahydroxy flavone”, is a flavanol naturally found in seeds, fruits, and vegetables [1] and has gained popularity as a dietary supplement due to its antioxidant and anti-inflammatory activities [2]. These capacities are attributed to the presence of catechol and hydroxyl groups in its structure that can act as antioxidant pharmacophores with the optimal configuration for scavenging free radicals [3]. Despite these benefits, the therapeutic application of Q is limited because of its physicochemical instability, high hydrophobicity, and poor bioavailability. Moreover, oral drugs’ bioavailability is lower than that of other administration routes due to factors such as harsh gastrointestinal conditions, digestive and luminal enzymes or efflux transporters, and restricted solubility, among others [4]. As a result, Q is removed from circulation before it reaches the target organs [5]. In addition, Q dietary supplements usually require a daily dose ranging from 500–1000 mg [6].

Researchers have recently developed novel drug delivery systems with advantages over conventional dosage forms, including site-of-action targeting, systemic bioavailability enhancement, action time prolongation, and target site specificity [7]. These systems could overcome the limitations of oral drug administration. Therefore, an effective oral drug delivery system should promote solubility in the gastrointestinal tract, protect the drug against gastrointestinal barriers, and improve its penetration across intestinal mucus [8]. 

In this regard, mesoporous silica particles (MSPs) have revolutionized the investigation of controlled drug delivery systems. MSPs are silicon dioxide structures with high chemical and mechanical stability, variable size (50–200 nm), large surface area (700–1000 m^2^/g), and a structure of well-ordered internal pores (diameter: 2–6 nm; volume: 0.6–1 cm^3^/g) [9,10]. The porous structure of MSPs can be used to load bioactive therapeutic molecules [11] and protect them from degradation. Mesoporous materials lack drug release ability and have limited capability to carry drugs to targeted cells when they are unmodified [12]; nevertheless, the ease of modifying them post-synthesis, adding ionic or covalent ligands, allows them to be designed as highly targeted systems for therapeutic interventions [13]. This allows the release of drugs in a specific area of interest, avoiding their premature release at off-target sites [14]. Moreover, MSP modifications could be made by adding organic moieties that can act as the gatekeeper in controlling the release of molecules via light, pH, ultrasound, thermal, redox, and other stimuli [15]. MSPs may also be used as matrices to enhance poorly water-soluble drugs’ solubility and dissolution rate [16]. The loading of bioactive molecules in mesoporous materials, such as α-tocopherol, curcumin, oligo phenol, and trans-β-carotene, among others [17], suggests their use as carriers for Q, too. In contrast with other types of particles used to encapsulate Q (proteins, liposomes, cyclodextrins, chitosan), silica-based microparticles have advantages like mechanical and chemical stability, biocompatibility, biodegradability, and low toxicity [18]. Q administration using MSPs has offered promising results in dermal formulations [19,20], in the prevention of bacterial infections in fish [21], and in targeted anticancer treatments [22,23,24], among others. Mesoporous silica materials could be applied to load Q to increase its aqueous solubility and absorbability, ultimately improving the bioavailability [25]. It should be noted that most of these studies do not use the oral administration route. 

Mesoporous silica has been reported to be safe for oral administration without significant adverse events or safety concerns, being mainly eliminated from the body without transformation [26]. Toxicity of mesoporous silica nanoparticles is dependent on size, dose, and cellular type; additionally, they are less toxic that non-porous silica nanoparticles, and surface functionalization reduces toxicity most likely due to reduction of surface density of silanol groups [27]. In its macro-form, the US Food and Drug Administration considers silica “generally recognized as safe (GRAS)” and nanoparticles of any size have not yet received regulatory approval. However, several clinical trials using silica administered using a variety of routes did not report any severe adverse reactions [28]. For food applications, silica-based materials produced and employed using the current hygiene standards and recommendations of use have no associated environmental or health risks [29]. The European Food Safety Authority consider that no further nano-specific testing would be needed for materials of particle size <500 nm showing <10% of particles smaller than 250 nm [30]. The study of MSPs as carriers of diverse drugs is extensive, but research about the use of MSPs to increase the bioaccessibility of Q as a dietary supplement is very limited, and to the best of our knowledge, there is a lack of reports testing MSPs to deliver Q using in vitro simulated gastrointestinal experiments.

This study aims to elucidate the effect of different chemical modifications (i.e., amine, carboxyl, or thiol group addition) on MSP performance as a Q delivery system under simulated gastrointestinal conditions. 

MSPs functionalized with simple moieties like carboxyl, amine, or thiol groups produce changes in electrostatic interactions with Q and modify its physical properties as a function of the pH of the simulated gastrointestinal medium. The interactions between Q and the different functional groups of MSPs define their characteristics as carriers; therefore, the degree of surface functionalization could be used to modulate the releasing properties as desired. 

This research demonstrates that the competence of a Q delivery system based on MSPs depends largely on its surface chemistry. MSP amine functionalization showed promissory effects such as an increment of Q-loading capacity and aqueous solubility and protection of its antioxidant capacity during gastric digestion. However, it also shows inferior characteristics important for cell internalization, such as particle size and Q release at physiological pH, where carboxyl and thiol functionalization showed better results. The tested MSP functionalizations provided satisfactory features for their use as a QODS.

## 2. Materials and Methods

### 2.1. Materials

Cetyltrimethylammonium bromide (CTAB), tetraethylorthosilicate (TEOS), 3-aminopropyltriethoxysilane (APTES), succinic anhydride (SA), mercaptopropyltrimethoxysilane (MPTMS), 12 kDa cut off dialysis membranes, quercetin dehydrate, ethanol (>99%), ethyl acetate, pepsin from porcine gastric mucosa (924 units/mg protein), pancreatin from porcine pancreas (activity = 4× USP), bile salts, NaHCO_3_, MgCl_2_(H_2_O)_6_ and (NH_4_)CO_3_, potassium persulfate, and 2,2′-azino-bis(3-ethylbenzothiazoline-6-sulfonic acid (ABTS) were purchased from Sigma-Aldrich (St. Louis, MO, USA). NaCl, KCl, Na_2_HPO_4_, and KH_2_PO_4_ were used for phosphate buffer saline (PBS) preparation and were acquired from Sigma-Aldrich. 

### 2.2. Synthesis and Functionalization of Mesoporous Silica Particles (MSPs)

The MSPs were synthesized according to [31], with modifications. A solution was prepared by dissolving 0.5 g of CTAB in 240 mL of distilled water, then 1.75 mL of 2 N NaOH was added, followed by heating to 80 °C. Subsequently, 2.5 mL of TEOS was added dropwise, under vigorous stirring. Once the TEOS was added, the stirring and temperature were maintained for two hours. The resultant product was filtered, washed twice with distilled water and ethanol, and dried overnight at room temperature before calcination at 500 °C for five hours.

To obtain functionalized MSPs (F-MSP) with amine, carboxyl, or thiol groups (A-MSP, C-MSP, and T-MSP, respectively), different types of functionalizing agents (F) were used, following Zaharudin et al.’s [22] methodology. Different F:MSP (*v*:*v*) ratios were tested to determine if MSP properties depend on the extent of the functionalized surface. For this purpose, a bifactorial experimental design was applied using “type of functionalizing agent” and “F:MSP ratio” as factors in three (APTES, SA, MPTMS) and two (high, low) levels, respectively. This experimental design was used only for FTIR spectroscopy analysis, loading capacity, kinetics of release, particle size, polydispersity index, and ζ potential determinations. For further experiments, only the “type of functionalizing agent” was used as a factor, and all F-MSPs were functionalized with a high F-MSP ratio. High and low levels were achieved for A-MSP production using 1 mL or 0.1 mL of APTES by dissolving in 20 mL of ethanol and adding 100 mg of MSPs. The particles were incubated under stirring for 24 h at room temperature and filtered and washed twice with ethanol. The same methodology was used for T-MSP obtention, replacing APTES with MPTMS. For C-MSP production, 100 mg of A-MSP was suspended in 50 mL of succinic anhydride in chloroform solution (2% *w*/*v* or 0.2% *w*/*v* for high and low levels, respectively). After 24 h of incubation under stirring, the particles were filtered and washed with chloroform, deionized water, and ethanol.

### 2.3. Physicochemical Characterization of F-MSPs

#### 2.3.1. FTIR Spectroscopy

FTIR spectra of F-MSPs were obtained in Horiba Jobin Yvon LabRAM IR2 (Kyoto, Japan) equipment in the 4000–600 cm^−1^ region with a resolution of 4 cm^−1^.

#### 2.3.2. Loading Capacity

For Q loading, 100 mg of particles were dispersed in 50 mL of Q dissolved in ethanol (2 mg/mL) and kept under stirring at room temperature until complete volatilization of the solvent. Then, MSPs loaded with Q (MSP/Q) were re-suspended in ethanol (50 mL) to remove residual Q and finally recovered by centrifugation at 2200× *g* for 20 min (Eppendorf 5804 R, Hamburg, Germany). The loading capacity was determined by measuring the residual Q concentration in the ethanol washings and using Equation (1):

Loading capacity (%) = ((Q_S_ − Q_R_)/M)) × 100 (1)

Q_S_ is the amount of Q in the initial ethanol solution, Q_R_ is the amount of Q in the washing ethanol, and M is the amount of F-MSPs.

#### 2.3.3. Particle Size, Polydispersity Index, and ζ Potential

The dynamic light scattering method measured the particle size, polydispersity index, and ζ potential of F-MSPs using ZetaSizer nano equipment (Malvern, Worcestershire, UK). Characterization was carried out for both bare F-MSP and those loaded with Q. For the analysis, the particles were previously suspended in PBS (1 mg/mL), adjusted to a pH of 1.5 or 7.4 (using 1 N HCl or 1 N NaOH), by using a 650 W ultrasonic homogenizer (Cole-Palmer, Vernon Hills, IL, USA) with a 3 mm microtip and 25% amplitude, for 10 s.

#### 2.3.4. Gas Physisorption Analysis

Pore size, volume, and surface area were determined for F-MSP loaded with Q by nitrogen physisorption analysis using the adsorption/desorption isotherms from an automated gas sorption analyzer (Autosorb iQ3, Quantachrome, Boynton Beac, FL, USA) at 77 K. All types of MSP were degassed at 180 °C for two hours under high-vacuum conditions. The surface area was obtained by the Brunauer–Emmett–Teller method, while pore volume and pore size distribution were obtained by the Barret–Joyner–Halenda method [32].

### 2.4. Quercetin In Vitro Release from F-MSP

The kinetics of Q release from F-MSP was determined according to Shao et al. [33], with modifications. First, 50 mg of MSPs loaded with Q were suspended in 10 mL of PBS in a dialysis bag (molecular weight cut off: 12 kDa, Sigma-Aldrich). Then, the dialysis bag was suspended in 90 mL of PBS under stirring (250 rpm) at 37 °C in the dark. One milliliter of sample was collected at intervals and replaced with one milliliter of fresh PBS to maintain constant volume release. The release media was previously adjusted to pH 1.5 or 7.4 to simulate gastric and intestinal pH. Drug release was measured in a Genesys 10 UV–Vis spectrophotometer (Thermo Scientific, Waltham, MA, USA) at 375 nm. The amount of drug released was calculated using Equation (2) [23]:

Cumulative release (%) = (Q_t_/Q_0_) × 100 (2)

Q_t_ is the Q in the release medium at t time, and Q_0_ is the Q loaded in the F-MSP.

To facilitate analysis of the kinetics of Q release, data were adjusted to a first-order model, following Equation (3):

Log Q_t_ = Log Q_0_ − (k_1_ t/2.303) (3)

where k_1_ is the first-order drug release rate constant, and Q_t_ is the amount of drug released at time t.

### 2.5. Simulated Gastrointestinal Digestion of Q-Loaded F-MSP

The static in vitro simulation of gastrointestinal food digestion was performed following [34], with slight modifications. The samples were prepared by dispersing 50 mg of particles in 11 mL of deionized water using an ultrasonic bath (Branson, Mod. 5510, Fisher Scientific, Waltham, MA, USA), and the control was 1 mg/mL of a Q suspension. 

Before simulated digestion, 1 mL aliquots were taken from the suspensions and centrifuged at 2200× *g* for 20 min. The effect of loading Q in F-MSP on its water solubility was determined by measuring the absorbance of supernatants at 375 nm and comparing it with that of a suspension of free Q, which was used as a control. The solubility of Q released from F-MSP relative to free Q was determined by using Equation (4):

Relative Q solubility (%) = (A_MSP_/A_FQ_) × 100 (4)

where A_MSP_ is the samples’ absorbance, and A_FQ_ is free Q control absorbance.

To start simulated gastric digestion (SGD), equal volumes of simulated gastric fluid and the suspensions were employed, and the mixture was adjusted to pH 3.0, using 1 N HCl. Following the Infogest method, simulated gastric fluid was a compound of an electrolyte solution and porcine pepsin diluted in water. The mixture was incubated at 37 °C, 200 rpm, in the dark, for two hours starting upon pepsin addition. Subsequently, the simulated intestinal digestion (SID) was conducted by adding 20 mL of simulated intestinal fluid (aqueous solution of salts, pancreatin, and bile salts). The mixture was adjusted to pH 7.0 using 1 N NaOH and then incubated for 2.5 h after pancreatin addition with the same conditions described for SGD. After both SGD and SID, antioxidant capacity and the concentration of Q in each simulated digestion medium (SDM) were determined. An ABTS radical solution was obtained by mixing 5 mL of ABTS (7.0 mM) and 88 µL of potassium persulfate (140 mM). After 16 h of contact time in the dark and refrigerated conditions, the solution was adjusted to an A_734 nm_ of 0.6–0.8 by adding absolute ethanol. Subsequently, 80 µL samples from SDM mixtures were added to 920 µL of adjusted ABTS radical solution. After a reaction time of 6 min, the A_734_ was measured; SGD and SID mixtures without MSP were used as controls. The ABTS radical scavenging ratio was calculated using Equation (5) [35]:

ABTS radical scavenging ratio (%) = (1 − A_1_/A_0_) × 100 (5)

A_0_ is the blank (water) absorbance, and A_1_ is the sample absorbance at 734 nm.

The Q concentration was determined following [36]. Aliquots of 1 mL were centrifuged at 2200× *g* for 20 min, then 400 µL of the supernatants were added to 3 mL of extracting solution (ethyl acetate and ethanol; 10:1 *v*/*v*). After vigorous stirring for 10 s, the mixtures were placed quiescently for 20 min to allow lamination (separation of the aqueous and organic phases). The Q content was determined by spectrophotometry using a quercetin standard curve (R^2^ > 0.999).

### 2.6. Statistical Analysis

All determinations were conducted using three independent replicates, except for the kinetics of the Q release experiment, and the mean ± standard deviation was reported. All comparisons were performed using the Tukey test (*p* < 0.05). For the experimental designs described, an analysis of variance was carried out. All comparisons were conducted using the Tukey test (*p* < 0.05).

## 3. Results and Discussion

### 3.1. Physicochemical Characterization of F-MSP

#### 3.1.1. FTIR Spectroscopy

**Figure 1:**
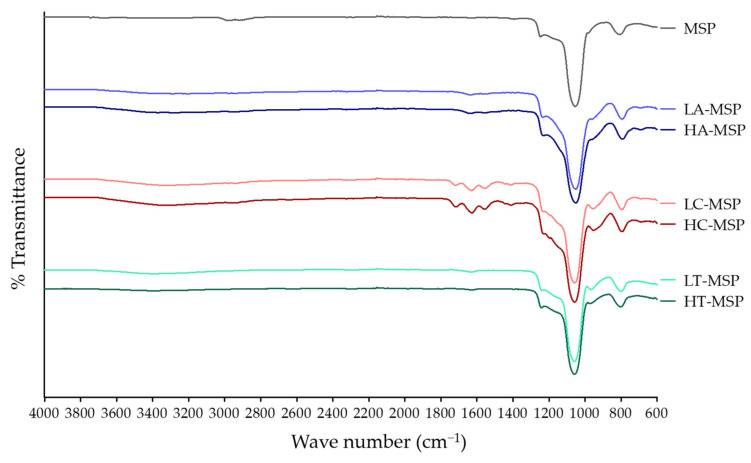
FTIR spectroscopy of F-MSPs. Treatments with low or high F:MSP ratio are represented with an L or an H before the functionalized MSP where, for functionalizations, A indicates the amine, C carboxyl, and T thiol group.

F-MSP was analyzed by FTIR spectroscopy (Figure 1) to ensure the presence of amine, carboxyl, or thiol functional groups. MSPs showed bands at 1067 cm^−1^ and 796 cm^−1^, corresponding to symmetric and asymmetric vibration of Si-O-Si bonding [24]. A-MSPs showed bands ranging from 1465 cm^−1^ to 1642 cm^−1^, attributed to the N-H group stretching [37]. C-MSPs showed a band at 1724 cm^−1^ assigned to the bending of the C=O bond of the carboxyl group, and the 1560 cm^−1^ and 1402 cm^−1^ bands were assigned to the symmetric and asymmetric vibration of the carboxyl group (-COO^−^) [38]. The characteristic bands corresponding to COOH and NH_2_ groups were found in F-MSP treated with low and high F:MSP ratios. On the other hand, modifications with thiol groups can be confirmed by a band at 2360 cm^−1^ [39], but T-MSP did not exhibit any band at this wave number. Further experiments are necessary to determine the presence of the thiol group.

#### 3.1.2. Loading Capacity

A-MSP showed the highest Q-loading capacities (19.79% ± 2.42% and 18.69% ± 1.87%, for HA-MSP and LA-MSP, respectively). These findings exhibit that the amine functionalization of MSPs about doubles (*p* < 0.05) its Q-loading capacity (9.92% ± 1.32%). 

This effect could be attributed to a better hydrogen-bonding interaction between A-MSP surface amine and Q hydroxyl groups [40]. Conversely, the carboxyl functionalization of MSP did not modify its loading capacity. At the same time, C-MSP showed values of 10.88% ± 1.81% and 7.88% ± 1.98% (for HC-MSP and LC-MSP, respectively), displaying loading capacities similar to MSPs (*p* < 0.05). 

There were no significant differences between high and low levels of F:MSP ratio for A-MSP and C-MSP loading capacities. However, the low to high F:MSP ratio for T-MSP increased the loading capacity from 5.94% ± 1.30% to 12.41 ± 1.32 (similar to MSPs). This could be related to the substitution of -OH in silanol groups by -SH groups that, due to the low electronegativity difference between S and H, form weaker hydrogen bonds with Q and, thus, when the surface density of -SH increases, this interaction is improved. 

#### 3.1.3. Particle Size, Polydispersity Index, and ζ Potential

MSP in vivo biodistribution and clearance are affected significantly by properties such as particle size and surface chemistry [41]. Particle size (PS), polydispersity index (PI), and ζ potential of F-MSP suspended in PBS adjusted to pH 1.5 or 7.4 are shown in Table 1. At pH 1.5, bare MSPs and F-MSP showed a PS > 1.5 µm except for A-MSP. The latter could be explained by the positive charge of A-MSP provided by protonated amine groups leading to particle repulsion, generating high ζ potential values (>30 mV). When A-MSPs are loaded with Q, their ζ potential decreases (<16 mV), leading to agglomerate formation and the increase in their PS (>1.8 µm). This observation could be related to decreased available NH_3_^+^ groups, suggesting a strong interaction between Q and A-MSPs through hydrogen bonds. Finally, all types of MSP loaded with Q exhibited a PS > 1.5 µm. This effect could be associated with the formation of hydrophobic interaction between particles loaded with Q.

On the other hand, at pH 7.4, C-MSP (HC or LC) showed the lowest PS of about 200 nm. This could be due to the partial deprotonation of the carboxyl groups, generating a negative net charge with the consequent particle repulsion (|ζ potential| > 38 mV). 

The PS of the C-MSPs may be suitable for their cell internalization in agreement with [42], who found that a particle diameter of 179 nm showed improved cell uptake using MDA-MB-231 and MCF10A cell lines compared with particles of 367 nm and 255 nm in diameter. PS also influences biodistribution: particles with a diameter greater than 200 nm activate the complement system and are quickly removed from the bloodstream, accumulating in the liver and spleen [43]. On the contrary, negatively charged particles have unfavorable interactions with membrane lipid and plasma proteins [15]. There was no significant difference (*p* < 0.05) in diameter between LC-MSP and HC-MSP or their ζ potential when they were loaded with Q, suggesting that carboxyl groups do not interact with Q. Supporting this statement is the fact that the loading capacity of C-MSP is equal to that of MSPs. 

T-MSP showed PS > 0.5 µm regardless of pH, DF, and loading state (bare or loaded with Q). This could be related to formation of disulfide bonds between particles that also may explain the absence of the characteristic band SH band for -SH groups upon FTIR spectroscopy. It is known that particles with sizes > 0.5 µm enter phagocytic cells via phagocytosis pathways [44]. Nonetheless, T-MSP demonstrated similar physical properties to the non-functionalized MSPs. 

#### 3.1.4. Gas Physisorption Analysis

**Figure 2:**
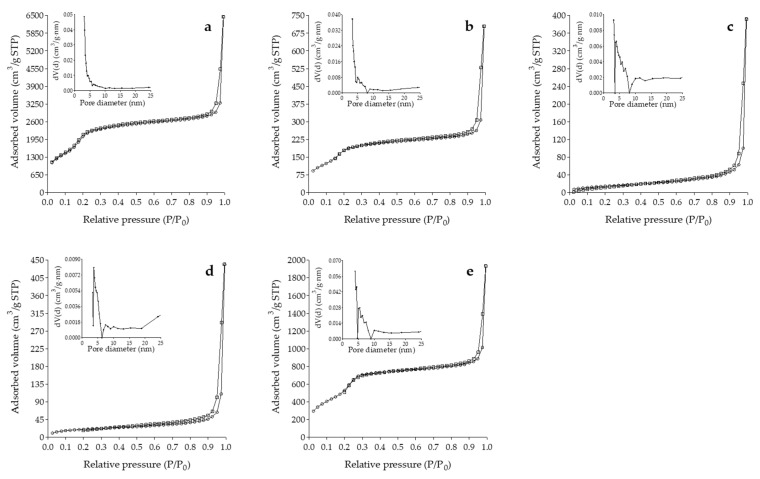
Nitrogen adsorption/desorption isotherms and pore size distribution curves (insets) of (**a**) MSPs, (**b**) MSP/Q, (**c**) A-MSP/Q, (**d**) C-MSP/Q, and (**e**) T-MSP/Q.

As can be seen in Figure 2, MSPs, MSP/Q, and T-MSP/Q showed type IV isotherms which are typical of mesoporous materials with homogeneously sized cylindrical pores [27] and show that the adsorption is conducted via multilayer adsorption followed by capillary condensation [45]. These materials also exhibit a capillary condensation step at low relative pressure (<0.3). On the other hand, A-MSP/Q and C-MSP/Q showed type III isotherms. Type III isotherms represent macroporous solids with multilayered adsorption, which probably resulted from the interspaces of small-sized particles that could also be evidenced by the hysteresis at high relative pressures [46]. For all samples loaded with Q, a significant decrease in N_2_ uptake was observed, indicating successful Q loading in the pores of the materials [47]. The H1 type hysteresis loop at high P/P_0_, which is characteristic of cylindrical pores open at both ends or of agglomerates of approximately spherical particles arranged in a reasonably regular array, is related to interparticle macroporosity [20]. 

MSPs showed a narrow size distribution (inset of Figure 2a). Still, for samples loaded with Q (insets of Figure 2b–e), two apparent reductions in volume adsorbed by pores in the range of 3–5 nm and 6–9 nm could be observed, which are likely related to the diameter of pores occupied by Q.

Textural parameters such as surface area, pore volume, and pore diameter of F-MSP are summarized in Table 2. S_BET_ of MSPs follows the literature [38]. MSP/Q showed a slight decrease in S_BET_ value, while A-MSP/Q and C-MSP/Q showed the lowest values. Significant reductions in the textural parameters, such as surface area and total pore volume of quercetin-loaded samples, indicate pore filling by quercetin [23].

A good delivery system should have a large enough pore size to facilitate the binding and diffusion of the molecules inside the pores [43]. It is reported that functionalization of MSP with amine, carboxyl, or thiol groups decreases pore volume and pore size due to the presence of additional grafted groups on the internal and external pore surface of the materials [39,47,48,49,50]. Drug loading also usually decreases pore volume and diameter [51,52]. Nevertheless, MSP/Q showed an increase in pore diameter and pore volume. 

This is likely related to the total filling of the smaller mesopores and the remaining small free volume of larger mesopores [53]. T-MSP showed the highest increase in textural parameters.

### 3.2. In Vitro Release of Q from F-MSP

**Figure 3:**
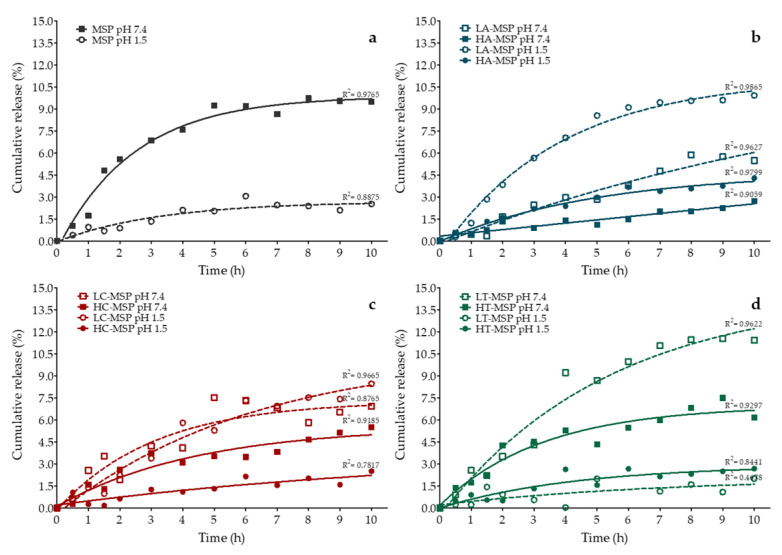
Kinetics of Q release in PBS at 37 °C from (**a**) MSPs, (**b**) A-MSPs, (**c**) C-MSPs, and (**d**) T-MSPs, suspended at pH 1.5 (circles) or 7.4 (squares). Treatments with low (discontinuous lines) or high (continuous lines) F:MSP ratio are represented with an L or an H, respectively, preceding the F-MSP alias. Lines refer to the first-order modeling with Equation (3), while symbols represent the experimental data.

To simulate the pH conditions experienced by F-MSPs loaded with Q throughout the gastrointestinal tract and conditions in the intracellular space, they were suspended in PBS adjusted to pH 1.5 and 7.4, respectively. Subsequently, the kinetics of Q release was monitored under these pH conditions (Figure 3). 

**Figure 4:**
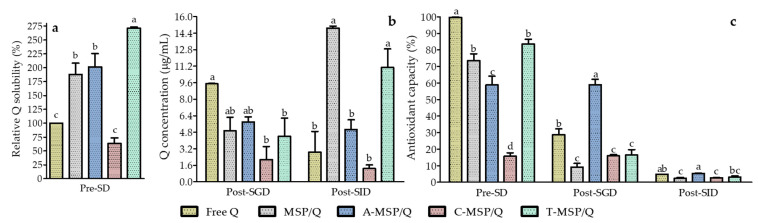
Relative Q solubility of F-MSP suspended in deionized water before simulated digestion (pre-SD) (**a**); and Q concentration (**b**); and antioxidant capacity (**c**) of simulated digestive medium containing F-MSP after simulated gastric digestion (Post-SGD) and after simulated intestinal digestion (Post-SID). In a column group, means (n = 2) not sharing the same letter are significantly different (*p* < 0.05).

The release of bioactive compounds from MSPs depends on the degree of interaction between them [47]. At pH 7.4, Q-loaded MSPs (Figure 4a) showed a maximum release of 9.71% after eight hours, while at pH 1.5, the release was slower, reaching a maximum release of 3.39% after 24 h. Quercetin pKa is around 6.3 [54], while the isoelectric point (pI) for MSPs is 2.0 [55]. In this regard, the accelerated release of Q at pH 7.4 could be due to repulsion forces between hydroxyl groups of the quercetin structure and the silanol groups on the MSP surface while both under this condition are partially deprotonated, providing a negative charge to the particles (ζ potential= −13.9 ± 0.85 mV). Also, an initial burst stage is associated with the fast transport of molecules precipitated on the surface, present on the pore opening, and loosely agglomerated inside the pores [52]. At pH 1.5, MSPs have a behavior closer to neutrality (ζ potential = 4.13 ± 2.01 mV), which allows retention of the bioactive compound by hydrogen bond formation. After ten hours, a decrease in Q concentration in the medium was noticed, which could be attributed to the low stability of Q in PBS media, where it undergoes a fast auto-oxidation process that reduces its half-life to approximately ten hours [56].

Meanwhile, A-MSP (Figure 3b) accelerated the release of Q when suspended at pH 1.5, indicating that the presence of electrically charged amine groups favors the drug’s release. This could be inconvenient, but an excellent pH-responsive delivery system is expected to limit premature release in acidic media (like gastric media) and show sustained release in intestine physiological conditions (pH 7.4) [4]. Also, it could be observed that A-MSP treated with a high F:MSP ratio showed improved drug retention. It was reported that functionalizing MSPs with aminopropyl groups using APTES could delay drug release by reducing the influx of liquid media in the pores and imparting some steric hindrance [57].

In turn, LC-MSP showed a faster release of Q than HC-MSP at pH 1.5 or 7.4 (Figure 3c). The Q release from LC-MSP was similar in both pH conditions. Finally, LT-MSP presented a faster Q release at pH 7.4, reaching a cumulative release of 11.43% after ten hours, compared to 2.00% at pH 1.5. This suggests that T-MSP could promote the release of Q in the intestine and within cells. The improved release of Q is associated with the large surface area showed by T-MSP that agrees with Hartono et al. [58], who observed that higher bioactive molecule release from mesoporous materials was presented by particles with a larger surface area.

### 3.3. Simulated Gastrointestinal Digestion of Q-Loaded F-MSP

The Q loaded in F-MSP modified the solubility in deionized water. This can be observed in Figure 4a, where T-MSP/Q exhibited the highest relative solubility, more than doubling (271.01% ± 2.45%) the Q solubility compared with free Q. MSP/Q and A-MSP/Q also increased Q solubility approximately twice (187.86% ± 20.44% and 201.73% ± 23.71%, respectively). According to AbouAitah et al. [57], when Q is loaded in MSPs, it is transformed to a non-crystalline state, modifying its solubility. In contrast, Q loaded in C-MSP did not show a solubility difference. 

Q concentration in the SDM was determined after simulated digestion’s gastric and intestinal phases (Figure 4b). After SGD, no significant differences were found between the concentration of free Q and that of Q released from MSPs and A-MSP. On the contrary, C-MSP and T-MSP appeared to delay the drug release. After SID, Q concentration in the media containing MSP/Q and T-MSP/Q was significantly (*p* < 0.05) higher compared with the media containing free Q, A-MSP/Q, and C-MSP/Q, which could be related to the promotion of drug release from the former materials in the intestinal pH conditions. These results could be explained by the pH-responsive Q release from F-MSP discussed in Section 3.2. 

Determination of antioxidant capacity (Figure 4c) shows that free Q can almost entirely inhibit ABTS radicals (99.71% ± 0.20%) before the simulated digestion. This capacity decreases drastically after SGD (28.80% ± 3.44%). This observation was similar for MSP/Q (antioxidant capacity decreased from 73.58% ± 4.15% to 9.17% ± 2.23%) and for T-MSP/Q (antioxidant capacity decreased from 83.67% ± 2.84% to 16.48% ± 3.24%).

Only A-MSP and C-MSP seemed to preserve the antioxidant capacity of Q in gastric media, but the higher Q content of A-MSP resulted in significant values before (58.88% ± 5.27%) and after SGD (58.95% ± 3.34%). This suggests that Q released from the carrier maintains more antioxidant activity than Q supplied in free form, likely due to the controlled Q release in this pH condition. Finally, after SID, all treatments exhibited deficient antioxidant capacity ranging from 5.38% ± 0.10% (A-MSP/Q) to 2.36% ± 0.63% (MSP/Q). These results suggest that despite the good release properties of MSPs and T-MSP in simulated intestinal media, all mesoporous materials failed to preserve the antioxidant properties of released Q. In aqueous medium at pH > 5, the ionized form of Q was reported as susceptible to degradation [59] and Q molecules are chemically unstable in the aqueous alkaline environment of the gastrointestinal tract because of possible reaction of the hydroxyl ions [60]. Continuous release of Q from MSPs and T-MSP resulted in a higher concentration of Q, which did not occur for the other treatments. However, it is probable that the Q released from the mesoporous materials, exposed to media conditions, generated products with decreased antioxidant activity, as previously reported [59,61,62]. Despite possible Q release degradation and loss of antioxidant capacity, the antioxidant effect may still be observed, since Q-derived free radicals could induce oxidative stress, increasing cellular production of superoxide anion. As a consequence, antioxidant cellular response is induced, leading to the reduction of total reactive oxygen substances (ROSs) [63]. It is relevant to highlight that the antioxidant capacity reported was that of Q released in the media after SGD and SID and did not involve the total amount of Q loaded into the MSPs. Considering that the nanomaterials provided controlled release of Q for up to 10 h, there could be within the particles a certain amount of Q that could be released after particles’ absorption and cell internalization. Major absorption takes place in the small intestine [64] through various segments [65], while the antioxidant capacity was measured after complete SID; therefore, Q released from A-MSP could exhibit antioxidant properties at early stages of intestinal digestion.

## 4. Conclusions

This study showed that the efficiency of a Q oral delivery system based on MSPs relies significantly on their surface chemistry. Q loaded in MSPs improved its aqueous solubility and conferred controlled release at gastric pH. Additionally, the tested MSP functionalizations provided satisfactory features for their use as a QODS: amine functionalization increased loading capacity and showed a protective effect of antioxidant properties of Q after gastric digestion. Carboxyl functionalization prevented agglomeration of particles, displaying suitable particle size for further cell endocytosis, while thiol functionalization offered the highest aqueous solubility and good release properties at physiological pH. Q loading in any mesoporous material did not provide antioxidant capacity protection when released under simulated intestinal conditions. This emphasizes the relevance of evaluating Q retention within MSPs after digestion and the assessment of cell uptake of Q-loaded MSPs that are necessary to elucidate if a QODS based on MSPs could improve its bioavailability.

## Figures and Tables

**Table 1:** Particle size (PS), polydispersity index (PI), and ζ potential of F-MSPs, bare or loaded with Q, suspended in PBS media at pH 1.5 or 7.4.

			PS (µm)	PI	ζ (mV)
Bare	pH 1.5	MSP	1.79 ± 0.22 ^bcCDE^	0.83 ± 0.16 ^abcAB^	1.57 ± 1.75 ^cdE^
		LA-MSP	0.69 ± 0.04 ^efF^	0.56 ± 0.05 ^cdeBCDE^	31.37 ± 0.70 ^aA^
		HA-MSP	0.87 ± 0.07 ^defF^	0.6 ± 0.04 ^bcdeBCDE^	32.93 ± 2.50 ^aA^
		LC-MSP	1.57 ± 0.06 ^bcdE^	0.54 ± 0.12 ^cdeCDE^	6.69 ± 1.36 ^bCD^
		HC-MSP	2.05 ± 0.15 ^bBC^	0.64 ± 0.09 ^abcdABCDE^	6.19 ± 0.997 ^bcCD^
		LT-MSP	1.95 ± 0.14 ^bBCD^	0.62 ± 0.11 ^bcdeBCDE^	1.31 ± 1.41 ^dE^
		HT-MSP	2.21 ± 0.14 ^bB^	0.64 ± 0.1 ^abcdBCDE^	1.62 ± 0.76 ^cdE^
	pH 7.4	MSP	1.03 ± 0.15 ^deDE^	0.63 ± 0.14 ^bcdeCD^	−15.83 ± 1.10 ^fBC^
		LA-MSP	3.93 ± 0.54 ^aA^	0.97 ± 0.06 ^aA^	−7.65 ± 1.86 ^eA^
		HA-MSP	3.33 ± 0.28 ^aAB^	0.86 ± 0.22 ^abcABC^	−6.71 ± 2.23 ^eA^
		LC-MSP	0.23 ± 0.004 ^fE^	0.34 ± 0.003 ^deEF^	−38 ± 1.95 ^gDE^
		HC-MSP	0.21 ± 0.01 ^fE^	0.3 ± 0.01 ^eF^	−44.1 ± 2.17 ^hF^
		LT-MSP	1.16 ± 0.41 ^cdeDE^	0.83 ± 0.13 ^abcABCD^	−14.23 ± 0.95 ^fBC^
		HT-MSP	1.92 ± 0.37 ^bCD^	0.92 ± 0.12 ^abAB^	−16.1 ± 0.57 ^fBC^
Loaded with Q	pH 1.5	MSP	1.62 ± 0.07 ^cdefDE^	0.64 ± 0.06 ^cdeBCDE^	4.13 ± 2.01 ^cDE^
		LA-MSP	1.86 ± 0.13 ^bcdCDE^	0.78 ± 0.1 ^bcABC^	13.93 ± 1.68 ^aB^
		HA-MSP	2.72 ± 0.12 ^bA^	0.92 ± 0.05 ^abA^	15.33 ± 0.31 ^aB^
		LC-MSP	1.63 ± 0.08 ^cdefDE^	0.48 ± 10 ^efDE^	8.85 ± 1.10 ^bC^
		HC-MSP	1.70 ± 0.03 ^cdeDE^	0.42 ± 0.03 ^fgE^	8.65 ± 1.39 ^bC^
		LT-MSP	1.79 ± 0.12 ^cdCDE^	0.64 ± 0.06 ^cdeBCDE^	1.22 ± 1.27 ^cE^
		HT-MSP	1.73 ± 0.12 ^cdeCDE^	0.73 ± 0.12 ^cdABCD^	1.66 ± 1.13 ^cE^
	pH 7.4	MSP	0.86 ± 0.03 ^efgDE^	0.66 ± 0.05 ^cdeBCD^	−13.9 ± 0.85 ^deBC^
		LA-MSP	2.43 ± 0.42 ^bcBC^	0.96 ± 0.06 ^abA^	−17.4 ± 1.71 ^eC^
		HA-MSP	3.96 ± 1.02 ^aA^	0.997 ± 0.01 ^aA^	−11.43 ± 1.02 ^dAB^
		LC-MSP	0.21 ± 0.004 ^gE^	0.24 ± 0.01 ^ghF^	−34.6 ± 0.20 ^fD^
		HC-MSP	0.21 ± 0.004 ^gE^	0.22 ± 0.01 ^hF^	−41.83 ± 3.09 ^gEF^
		LT-MSP	1.17 ± 0.005 ^defDE^	0.62 ± 0.06 ^cdeCD^	−16.87 ± 1.80 ^eC^
		HT-MSP	0.73 ± 0.03 ^fgE^	0.57 ± 0.04 ^defDE^	−14.57 ± 0.67 ^deBC^

The results shown are mean values with *n* = 3. For “Bare” or “Loaded with Q”, columns not sharing the same lowercase letter are significantly different (*p* < 0.05). For pH 1.5 or 7.4, values not sharing the same uppercase letter are significantly different (*p* < 0.05).

**Table 2:** The surface area (S_BET_), pore volume (VP), and pore diameter (DP) of F-MSPs loaded with Q.

	S_BET_ (m^2^/g)	V_P_ (cm^3^/g)	D_P_ (nm)
MSPs	871.508	0.659	3.136
MSP/Q	767.986	0.808	3.315
A-MSP/Q	56.480	0.606	3.136
C-MSP/Q	76.961	0.669	3.714
T-MSP/Q	2022.822	1.946	3.933

A-MSP/Q: A-MSP loaded with Q; C-MSP/Q: C-MSP loaded with Q; T-MSP/Q: T-MSP loaded with Q.

## Data Availability

The original contributions presented in the study are included in the article, further inquiries can be directed to the corresponding author.

## References

[1] D’Andrea G. (2015). Quercetin: A flavonol with multifaceted therapeutic applications?. Fitoterapia.

[2] Batiha G.E., Beshbishy A.M., Ikram M., Mulla Z.S., El-Hack M.E.A., Taha A.E., Algammal A.M., Elewa Y.H.A. (2020). The pharmacological activity, biochemical properties, and pharmacokinetics of the major natural polyphenolic flavonoid: Quercetin. Foods.

[3] Heijnen C.G., Haenen G.R., Oostveen R.M., Stalpers E.M., Bast A. (2002). Protection of flavonoids against lipid peroxidation: The structure-activity relationship revisited. Free Radic. Res..

[4] Florek J., Caillard R., Kleitz F. (2017). Evaluation of mesoporous silica nanoparticles for oral drug delivery—current status and perspective of MSNs drug carriers. Nanoscale.

[5] Moghadam M.K., Dorraji M.S.S., Dodangeh F., Ashjari H.R., Mousavi S.N., Rasoulifard M.H. (2022). Design of a new light curable starch-based hydrogel drug delivery system to improve the release rate of quercetin as a poorly water-soluble drug. Eur. J. Pharm. Sci..

[6] Andres S., Pevny S., Ziegenhagen R., Bakhiya N., Schäfer B., Hirsch-Ernst K.I., Lampen A. (2017). Safety aspects of the use of quercetin as a dietary supplement. Mol. Nutr. Food Res..

[7] Stephen S., Gorain B., Choudhury H., Chatterjee B. (2021). Exploring the role of mesoporous silica nanoparticle in the development of novel drug delivery systems. Drug Deliv. Transl. Res..

[8] Sreeharsha N., Philip M., Krishna S.S., Viswanad V., Sahu R.K., Shiroorkar P.N., Aasif A.H., Fattepur S., Asdaq S.M.B., Nair A.B. (2022). Multifunctional mesoporous silica nanoparticles for oral drug delivery. Coatings.

[9] Jafari S., Derakhshankhah H., Alaei L., Fattahi A., Varnamkhasti B.S., Saboury A.A. (2019). Mesoporous silica nanoparticles for therapeutic/diagnostic applications. Biomed. Pharmacother..

[10] Vallet-Regı M., Colilla M., Izquierdo-Barba I., Manzano M. (2017). Mesoporous silica nanoparticles for drug delivery: Current insights. Molecules.

[11] Thi T.T.H., Du Cao V., Nguyen T.N.Q., Hoang D.T., Ngo V.C., Nguyen D.H. (2019). Functionalized mesoporous silica nanoparticles and biomedical applications. Mat. Sci. Eng. C.

[12] Wibowo F.R., Saputra O.A., Lestari W.W., Koketsu M., Mukti R.R., Martien R. (2020). pH-triggered drug release controlled by poly(styrene sulfonate) growth hollow mesoporous silica nanoparticles. ACS Omega.

[13] Chen N., Cheng S., Souris J.S., Chen C., Mou C., Lo L. (2013). Theranostic applications of mesoporous silica nanoparticles and their organic/inorganic hybrids. J. Mater. Chem. B.

[14] Siddiqui B., Rehman A.U., Ul-Haq I., Al-Dossary A.A., ElaıSsari A., Ahmed N. (2022). Exploiting recent trends for the synthesis and surface functionalization of mesoporous silica nanoparticles towards biomedical applications. Int. J. Pharm. X.

[15] Saputra O.A., Lestari W.A., Kurniansyah V., Lestari W.W., Sugiura T., Mukti R.R., Martien R., Wibowo F.R. (2022). Organically surface engineered mesoporous silica nanoparticles control the release of quercetin by pH stimuli. Sci. Rep..

[16] Maleki A., Kettiger H., Schoubben A.M.M., Rosenholm J.M., Ambrogi V., Hamidi M. (2017). Mesoporous silica materials: From physico-chemical properties to enhanced dissolution of poorly water-soluble drugs. J. Control. Release.

[17] Bernardos A., Kouřimská L. (2013). Applications of mesoporous silica materials in food—A review. Czech J. Food Sci..

[18] Morante-Zarcero S., Endrino A., Casado N., Pérez-Quintanilla D., Sierra I. (2021). Evaluation of mesostructured silica materials with different structures and morphologies as carriers for quercetin and naringin encapsulation. J. Porous Mater..

[19] Popova M., Trendafilova I., Szegedi Á., Mihály J., Németh P., Marinova S.G., Aleksandrov H.A., Vayssilov G.N. (2016). Experimental and theoretical study of quercetin complexes formed on pure silica and Zn-modified mesoporous MCM-41 and SBA-16 materials. Microporous Mesoporous Mater..

[20] Berlier G., Gastaldi L., Ugazio E., Miletto I., Iliade P., Sapino S. (2013). Stabilization of quercetin flavonoid in MCM-41 mesoporous silica: Positive effect of surface functionalization. J. Colloid Interface Sci..

[21] Shabana N., Taju G., Majeed S., Ahmed A.N., Karthika M.S., Ramasubramanian V., Hameed A.S. (2021). Preparation and evaluation of mesoporous silica nanoparticles loaded quercetin against bacterial infections in *Oreochromis niloticus*. Aquac. Rep..

[22] Zaharudin N.S., Isa E.D.M., Ahmad H., Rahman M.B.A., Jumbri K. (2020). Functionalized mesoporous silica nanoparticles templated by pyridinium ionic liquid for hydrophilic and hydrophobic drug release application. J. Saudi Chem. Soc..

[23] Trendafilova I., Szegedi Á., Mihály J., Momekov G., Lihareva N., Popova M. (2017). Preparation of efficient quercetin delivery system on Zn-modified mesoporous SBA-15 silica carrier. Mater. Sci. Eng. C.

[24] Sarkar A., Ghosh S., Chowdhury S., Pandey B., Sil P.C. (2016). Targeted delivery of quercetin loaded mesoporous silica nanoparticles to the breast cancer cells. Biochim. Biophys. Acta.

[25] Wang Y., Tao B., Wan Y., Sun Y., Wang L., Sun J., Li C. (2020). Drug delivery based pharmacological enhancement and current insights of quercetin with therapeutic potential against oral diseases. Biomed. Pharmacother..

[26] Petrișor G., Motelică L., Ficai D., Truşcă R., Surdu V., Voicu G., Oprea O., Ficai A., Andronescu E. (2022). New mesoporous silica materials loaded with polyphenols: Caffeic acid, ferulic acid and p-coumaric acid as dietary supplements for oral administration. Materials.

[27] Garrido-Cano I., Candela-Noguera V., Herrera G., Cejalvo J.M., Lluch A., Marcos M.D., Sancenón F., Eroles P., Martínez-Máñez R. (2021). Biocompatibility and internalization assessment of bare and functionalised mesoporous silica nanoparticles. Microporous Mesoporous Mater..

[28] Tng D.J.H., Low J.G.H. (2023). Current status of silica-based nanoparticles as therapeutics and its potential as therapies against viruses. Antiviral Res..

[29] Ros-Lis J.V., Bernardos A., Pérez É., Baviera J.M.B., Martínez-Máñez R. (2018). Functionalized Silica Nanomaterials as a New Tool for New Industrial Applications.

[30] Schoonjans R., Castenmiller J., Chaudhry Q., Cubadda F., Daskaleros T., Franz R., Gott D.M., Mast J., Mortensen A., Oomen A.G. (2023). Regulatory safety assessment of nanoparticles for the food chain in Europe. Trends Food Sci. Technol..

[31] Fernandez-Bats I., Di Pierro P., Villalonga-Santana R., García-Almendárez B.E., Porta R. (2018). Bioactive mesoporous silica nanocomposite films obtained from native and transglutaminase-crosslinked bitter vetch proteins. Food Hydrocoll..

[32] Li J., Shen S., Kong F., Jiang T., Tang C., Chen Y. (2018). Effects of pore size on in vitro and in vivo anticancer efficacies of mesoporous silica nanoparticles. RSC Adv..

[33] Shao M., Chang C., Liu Z., Chen K., Zhou Y., Zheng G., Huang Z., Xu H., Xu P., Lü B. (2019). Polydopamine coated hollow mesoporous silica nanoparticles as pH-sensitive nanocarriers for overcoming multidrug resistance. Colloids Surf. B Biointerfaces.

[34] Brodkorb A., Egger L., Alminger M., Alvito P., Assunção R., Ballance S., Bohn T., Bourlieu-Lacanal C., Boutrou R., Carrière F. (2019). INFOGEST static in vitro simulation of gastrointestinal food digestion. Nat. Protoc..

[35] Liang Q., Sun X., Raza H., Khan M.A., Ma H., Ren X. (2021). Fabrication and characterization of quercetin loaded casein phosphopeptides-chitosan composite nanoparticles by ultrasound treatment: Factor optimization, formation mechanism, physicochemical stability and antioxidant activity. Ultrason. Sonochem..

[36] Chen F., Ou S., Tang C. (2016). Core–shell soy protein–soy polysaccharide complex (nano)particles as carriers for improved stability and sustained release of curcumin. J. Agric. Food Chem..

[37] Rumman G.A., Al-Musawi T.J., Sillanpää M., Balarak D. (2021). Adsorption performance of an amine-functionalized MCM–41 mesoporous silica nanoparticle system for ciprofloxacin removal. Environ. Nanotechnol. Monit. Manag..

[38] Gou K., Wang Y., Guo X., Wang Y., Bian Y., Zhao H., Guo Y., Pang Y., Xie L., Li S. (2021). Carboxyl-functionalized mesoporous silica nanoparticles for the controlled delivery of poorly water-soluble non-steroidal anti-inflammatory drugs. Acta Biomater..

[39] Velusamy P., Srinivasa C.M., Kumar G.V., Qurishi Y., Su C., Gopinath S.C.B. (2018). A pH stimuli thiol modified mesoporous silica nanoparticles: Doxorubicin carrier for cancer therapy. J. Taiwan Inst. Chem. Eng..

[40] Wang X., Li C., Fan N., Li J., Zhang H., Sun J. (2017). Multimodal nanoporous silica nanoparticles functionalized with aminopropyl groups for improving loading and controlled release of doxorubicin hydrochloride. Mater. Sci. Eng. C.

[41] Dogra P., Adolphi N.L., Wang Z., Yu L., Butler K.S., Durfee P.N., Croissant J.G., Noureddine A., Coker E.N., Bearer E.L. (2018). Establishing the effects of mesoporous silica nanoparticle properties on in vivo disposition using imaging-based pharmacokinetics. Nat. Commun..

[42] Mazzotta E., De Santo M., Lombardo D., Leggio A., Pasqua L. (2022). Mesoporous silicas in materials engineering: Nanodevices for bionanotechnologies. Mater. Today Bio.

[43] Saputra O.A., Wibowo F.R., Lestari W.W. (2022). High storage capacity of curcumin loaded onto hollow mesoporous silica nanoparticles prepared via improved hard-templating method optimized by Taguchi DoE. Eng. Sci. Technol. Int. J..

[44] Oh N., Park J. (2014). Endocytosis and exocytosis of nanoparticles in mammalian cells. Int. J. Nanomed..

[45] Kruk M., Jaroniec M. (2001). Gas Adsorption Characterization of ordered organic−inorganic nanocomposite materials. Chem. Mater..

[46] Chiang Y., Lian H., Leo S., Wang S., Yamauchi Y., Wu K.C. (2011). Controlling particle size and structural properties of mesoporous silica nanoparticles using the Taguchi method. J. Phys. Chem. C..

[47] Kamarudin N.H.N., Jalil A.A., Triwahyono S., Salleh N.F.M., Karim A.H., Mukti R.R., Hameed B., Ahmad A. (2013). Role of 3-aminopropyltriethoxysilane in the preparation of mesoporous silica nanoparticles for ibuprofen delivery: Effect on physicochemical properties. Microporous Mesoporous Mater..

[48] Tran V.A., Lee S. (2018). A prominent anchoring effect on the kinetic control of drug release from mesoporous silica nanoparticles (MSNs). J. Colloid Interface Sci..

[49] Popova M., Trendafilova I., Tsacheva I., Mitova V., Kyulavska M., Koseva N., Mihály J., Momekova D., Momekov G., Aleksandrov H.A. (2018). Amino-modified KIT-6 mesoporous silica/polymer composites for quercetin delivery: Experimental and theoretical approaches. Microporous Mesoporous Mater..

[50] Cheah W., Sim Y., Yeoh F. (2016). Amine-functionalized mesoporous silica for urea adsorption. Mater. Chem. Phys..

[51] Patiño-Herrera R., Louvier-Hernández J.F., Escamilla-Silva E.M., Chaumel J., Palestino G., Pérez E. (2019). Prolonged release of metformin by SiO_2_ nanoparticles pellets for type II diabetes control. Eur. J. Pharma. Sci..

[52] Szewczyk A., Prokopowicz M. (2020). Mesoporous silica pellets—A promising oral drug delivery system?. J. Drug Deliv. Sci. Technol..

[53] Bahrami Z., Badiei A., Atyabi F. (2014). Surface functionalization of SBA-15 nanorods for anticancer drug delivery. Chem. Eng. Res. Des..

[54] Pool H., Mendoza S., Xiao H. (2013). Encapsulation and release of hydrophobic bioactive components in nanoemulsion-based delivery systems: Impact of physical form on quercetin bioaccessibility. Food Funct..

[55] Lázaro A., Sato K., Brouwers H.J., Geus J. (2018). Pore structure development of silica particles below the isoelectric point. Microporous Mesoporous Mater..

[56] Kim M.K., Park K., Lee C., Park H., Choo H., Chong Y. (2010). Enhanced stability and intracellular accumulation of quercetin by protection of the chemically or metabolically susceptible hydroxyl groups with a pivaloxymethyl (POM) promoiety. J. Med. Chem..

[57] AbouAitah K., Farghali A.A. (2016). Mesoporous silica materials in drug delivery system: Ph/glutathione- responsive release of poorly water-soluble pro-drug quercetin from two and three-dimensional pore-structure nanoparticles. J. Nanomed. Nanotechnol..

[58] Hartono S.B., Hadisoewignyo L., Yang Y., Meka A.K., Antaresti, Yu C. (2016). Amine functionalized cubic mesoporous silica nanoparticles as an oral delivery system for curcumin bioavailability enhancement. Nanotechnology.

[59] Zheng Y., Haworth I.S., Zuo Z., Chow M.S., Chow A.H. (2005). Physicochemical and structural characterization of quercetin-β-cyclodextrin complexes. J. Pharm. Sci..

[60] Dey M., Ghosh B., Giri T.K. (2020). Enhanced intestinal stability and pH sensitive release of quercetin in GIT through gellan gum hydrogels. Colloids Surf. B Biointerfaces..

[61] Zvezdanović J., Stanojevic J., Marković D., Cvetković D. (2012). Irreversible UV-induced quercetin and rutin degradation in solution, studied by UV-spectrophotometry and HPLC chromatography. J. Serb. Chem. Soc..

[62] Jurasekova Z., Domingo C., García-Ramos J.V., Sánchez-Cortés S. (2014). Effect of pH on the chemical modification of quercetin and structurally related flavonoids characterized by optical (UV-visible and Raman) spectroscopy. Phys. Chem. Chem. Phys..

[63] Carrillo-Garmendia A., Madrigal-Pérez L.A., Regalado-González C. (2023). The multifaceted role of quercetin derived from its mitochondrial mechanism. Mol. Cell. Biochem..

[64] Yu H., Zhang Y., Liang Y., Ma X., Xiao Q., Xiao J., Wang X., Luo Y., Yue T. (2020). Advance on the absorption, metabolism, and efficacy exertion of quercetin and its important derivatives. Food Frontiers..

[65] Li H., Zhao X., Ma Y., Zhai G., Li L., Lou H. (2009). Enhancement of gastrointestinal absorption of quercetin by solid lipid nanoparticles. J. Control. Release..

